# Numerical Investigation of Heat Transfer and Friction Factor Characteristics in a Circular Tube Fitted with V-Cut Twisted Tape Inserts

**DOI:** 10.1155/2013/492762

**Published:** 2013-09-01

**Authors:** Sami D. Salman, Abdul Amir H. Kadhum, Mohd S. Takriff, Abu Bakar Mohamad

**Affiliations:** ^1^Department of Chemical and Process Engineering, Faculty of Engineering and Built Environment, Universiti Kebangsaan Malaysia, 43600 Bangi, Selangor, Malaysia; ^2^Biochemical Engineering Department, Al-Khwarizmi College of Engineering, University of Baghdad, Baghdad 47024, Iraq

## Abstract

Numerical investigation of the heat transfer and friction factor characteristics of a circular fitted with V-cut twisted tape (VCT) insert with twist ratio (y = 2.93) and different cut depths (w = 0.5, 1, and 1.5 cm) were studied for laminar flow using CFD package (FLUENT-6.3.26). The data obtained from plain tube were verified with the literature correlation to ensure the validation of simulation results. Classical twisted tape (CTT) with different twist ratios (y = 2.93, 3.91, 4.89) were also studied for comparison. The results show that the enhancement of heat transfer rate induced by the classical and V-cut twisted tape inserts increases with the Reynolds number and decreases with twist ratio. The results also revealed that the V-cut twisted tape with twist ratio y = 2.93 and cut depth w = 0.5 cm offered higher heat transfer rate with significant increases in friction factor than other tapes. In addition the results of V-cut twist tape compared with experimental and simulated data of right-left helical tape inserts (RLT), it is found that the V-cut twist tape offered better thermal contact between the surface and the fluid which ultimately leads to a high heat transfer coefficient. Consequently, 107% of maximum heat transfer was obtained by using this configuration.

## 1. Introduction

Heat exchangers with the convective heat transfer are widely used in many engineering applications. Enhancement of heat transfer in all types of thermotechnical apparatus is of great significance for the industry. Besides the savings of primary energy, it also leads to a reduction in size and weight. In general, heat transfer enhancement techniques can be divided into two categories: (1) active techniques which need external power source and  (2) passive techniques which do not need external power source. Several experimental studies on heat augmentation techniques using twisted tape as passive technique have been reported in the literature [1–15]. Thereafter and due to advances in computer hardware and software and consequent increase in calculation speed, the CFD modeling technique was developed as a powerful and effective tool for better understanding of the complex hydrodynamics in many industrial processes. Kumar et al. [16] studied the hydrodynamics and heat transfer characteristics of tube in the form of the helical heat exchanger at a pilot plant scale fitted with semicircular plate in annuals area using a commercial CFD package to predict the flow and thermal properties in a tube of the helical heat exchanger. They found that the results of the simulated data of the Nusselt number and friction factor in the inner and outer tubes coincide with experimental data. Sivashanmugam et al. [17] presented the modeling of heat transfer augmentation in a circular tube fitted with a helical twist insert in a laminar and turbulent flow using CFD. Jayakumar et al. [18] offered a comparison study of CFD simulations to experiment on convective heat transfer in double pipe helical heat exchanger and developed an empirical correlation for estimation of the inner heat transfer coefficient of a helical coil. Lei et al. [19] conducted the hydrodynamics and heat transfer study on heat exchanger with a single-segment baffle, single-layer helical baffle, and two-layer helical baffle experimentally as well as numerically using the CFD method. They found that the configuration of helical baffles gives a higher heat transfer coefficient than a single-layer baffle at the same pressure drop and the configuration of a two-layer helical baffle has better heat performance than that of the single-layer baffle. Kharat et al. [20] developed a new correlation of heat transfer coefficient between concentric helical coils of the heat exchanger which depended on experimental work and CFD simulation. FLUENT 6.3.26 has been used to improve the heat transfer coefficient correlation for the flue gas side to optimize the gap between concentric coils. Nagarajan and Sivashanmugam [21] presented a simulation of heat transfer augmentation and friction factor characteristics of a circular tube fitted with a right-left helical twist insert with a 100 mm spacer using CFD. The results of a simulated Nusselt number, friction factor for a given flow rate, and twist ratio were compared with the experimental data and showed a good agreement. Shabanian et al. [22] conducted an experiment and CFD modeling studies on heat transfer, friction factor, and thermal performance of an air-cooled heat exchanger equipped with three types of tube insert including butterfly, classic, and jagged twisted tapes. They found that the predicted results in terms of turbulence intensity have a good agreement with measured values of Nu number and friction factor. Wang et al. [23] explicated the optimum configuration of regularly spaced short-length twisted tape in a circular tube by using computational fluid dynamics (CFD) modeling. The air was used as the test fluid with a turbulent flow with the configuration parameters including the free space ratio (*s*), twist ratio (*y*), and rotated angle (*a*). The predicted results were in a good agreement with experimental data. The results showed that the larger rotated angle yields a higher heat transfer value and greater flow resistance, whereas the smaller twist ratio resulted in better heat transfer performance except for a larger rotated angle at a high Reynolds number. Pathipakka and Sivashanmugam [12] proposed CFD simulation of the heat transfer characteristics of Al_2_O_3_ nanofluid in a circular tube fitted with helical twist inserts under constant heat flux using FLUENT version 6.3.26 in a laminar flow. The Al_2_O_3_ nanoparticles in water at different concentrations (0.5%, 1.0%, and 1.5%) and helical twist inserts with different twist ratios (*y* = 2.93, 3.91, and 4.89) were used for the simulation. The data obtained by simulation were compared with the literature value of water for plain tube helical tape inserts. Salman et al. [24] report an application of a mathematical model of the heat transfer enhancement and friction factor characteristics of water in constant heat-fluxed tube fitted with elliptical cut twisted tape inserts with twist ratios (*y* = 2.93, 3.91, and 4.89) and different cut depths (*w* = 0.5, 1, and 1.5 cm) under laminar flow using FLUENT version 6.3.26. The results elaborated that the enhancement of heat transfer rate and the friction factor induced by elliptical cut twisted tape inserts increases with the Reynolds number and decreases with twist ratio. In addition the results show that the elliptical cut twisted tape with twist ratio *y* = 2.93 and cut depth *w* = 0.5 cm offered higher heat transfer rate with significant increases in friction factor. In the present work a numerical investigation of heat transfer enhancement in a tube induced by V-cut twist tape inserts is reported using CFD simulation based on experimental work mentioned in [12]. The result obtained by this configuration enhanced the heat transfer rate than those obtained by classical and helical twist inserts. This study can be used as guideline for experimental work of heat transfer augmentation.

## 2. Technical Details 

**Figure 1:**
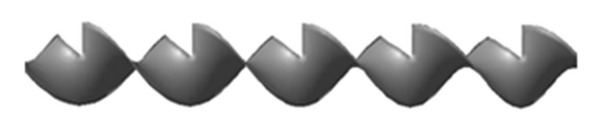
V-cut twisted tape insert.

The geometrical configuration of V-cut twisted tape (VCT) inserts with a thickness (*t*) 0.08 cm, width (*W*) of 2.45 cm, length (*L*) of 180 cm with relative twisted ratios (*y* = 2.93, 3.91, and 4.89), and cut depth (*w* = 0.5, 1 and 1.5 cm) is used for simulation. This configuration is illustrated in Figure 1. 

## 3. Physical Model

**Figure 2:**
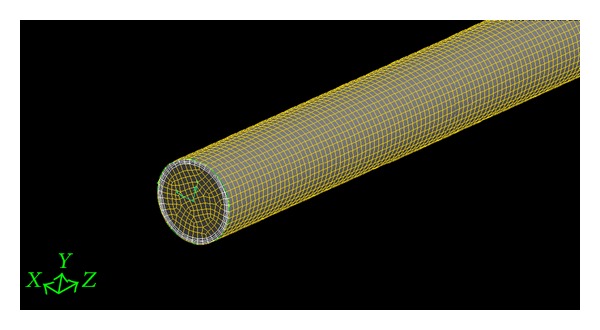
Grid for the plain tube.

**Figure 3:**
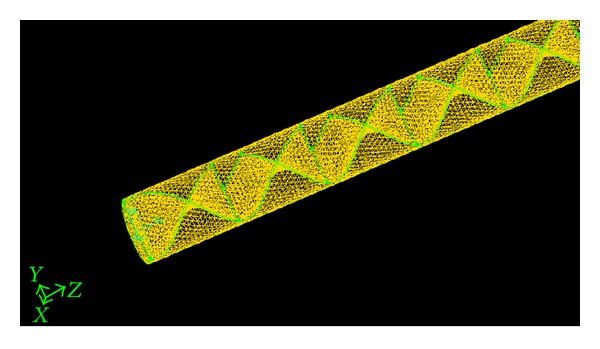
Grid for the plain tube fitted with V-cut twisted (VCT) tape insert.

**Figure 4:**
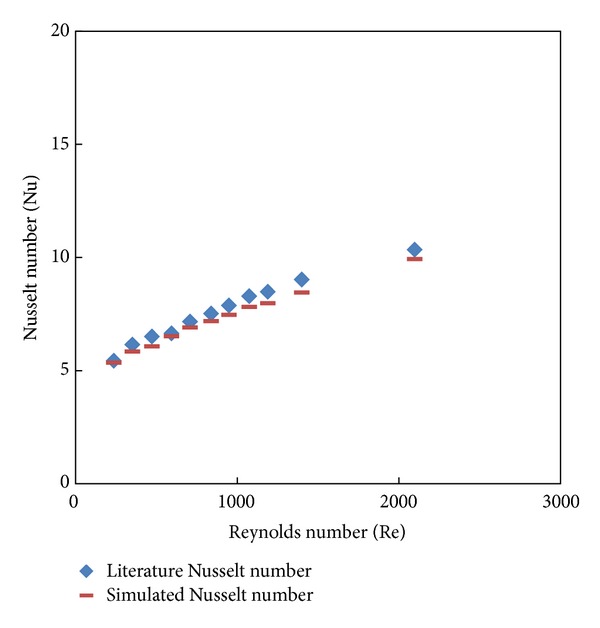
Plain tube simulated Nusselt number versus the literature data.

The GAMBIT program is used for generation of the geometry and grids whereas FLUENT version 6.3.26 is used for module preprocessing. The geometry and the grid of the plain tube and V-cut twisted tape inserts are as shown in Figures 2 and 3, and these configurations were created in GAMBIT and imported into FLUENT for simulation. The geometry of classical and V-cut twisted tape inserts was made by winding a uniform strip of 25.54 mm width using the twist option in the sweeping of faces. The twist angle of 360° with a length of 75, 100, and 125 mm for various twist ratios (*y* = 2.93, 3.91, and 4) was generated by using a perpendicular type of sweeping for the entire length of 1800 mm. The volume required for simulation was created by the subtraction of the twisted tape insert from the plain tube geometry. The edged meshes were applied to each edge by using the particular interval count, whereas the front circular face was mashed by using tetrahedral and pave type meshing. The mesh face was swept over the entire volume using tetrahedral/hybrid elements and a T grid type. Boundary conditions for the mesh volume were inlet, outlet, wall, and type of fluid defined. Subsequently, the mesh file was exported to FLUENT for simulation. The following equations used to calculate the Nsselt number (Nu) and the skin friction factor (*f*) (see Figure 4).

(1) Nu=hDK,h=q∙Tw−Tb,f=16Re,Re=ρuDμ,

where *D* is the tube diameter, *h* is the heat transfer coefficient, *k* is the conductivity of water, *q* ^∙^ is the heat flux on the tube, *T*_*w*_ is the tube wall temperature, and *T*_*b*_ is the bulk temperature of water *T*_*b*_ = (*T*_*o*_ + *T*_*i*_)/2. *ρ* is the density, *μ* is dynamic viscosity, and *u* is the water velocity. 

## 4. Numerical Simulations

The problem investigated is a three-dimensional steady state laminar flow through a plain tube fitted with classical and V-cut twisted tape inserts under constant heat-fluxed tube using the following governing equations.

### 4.1. Continuity Equation for an Incompressible Fluid

(2) $$\begin{array}{c} \frac{\partial p}{\partial t}+\nabla \cdot \left(\rho \vec{\upsilon }\right)=S_{m}. \end{array}$$

### 4.2. Conservation of Momentum

(3) $$\begin{array}{c} \frac{\partial \upsilon }{\partial t}+\rho \left(\vec{\upsilon }\cdot \nabla \right)\vec{\upsilon }=-\nabla p+\rho \bar{g}+\nabla \cdot \tau_{ij}+\vec{F}. \end{array}$$

### 4.3. Conservation of Energy

(4) ρ∂∂t(ρE)+∇·{υ→(ρE+ρ)}  =∇·{Keff∇T−∑hi(τ→eff·υ→)}+Sh.

## 5. Modeling Parameters

The thermophysical properties of the fluid, materials, and the numerical values of the mass flow rate and heat flux which were used in a number of the simulations are given in Tables 1 and 2.

## 6. Numerical Method

The commercial package of CFD (FLUENT 6.3.26) was chosen as the CFD tool for this study to solve the above-mentioned governing equations accompanied with boundary conditions. Solution sequential algorithm (segregated solver algorithm) with settings including implicit formulation, steady (time-independent) calculation, viscous laminar model and energy equation, SIMPLE as the pressure-velocity coupling method, and first-order upwind scheme for energy and momentum equations was selected for simulation. 

## 7. Results and Discussion 

### 7.1. Grid Testing and Code Validation

**Figure 5:**
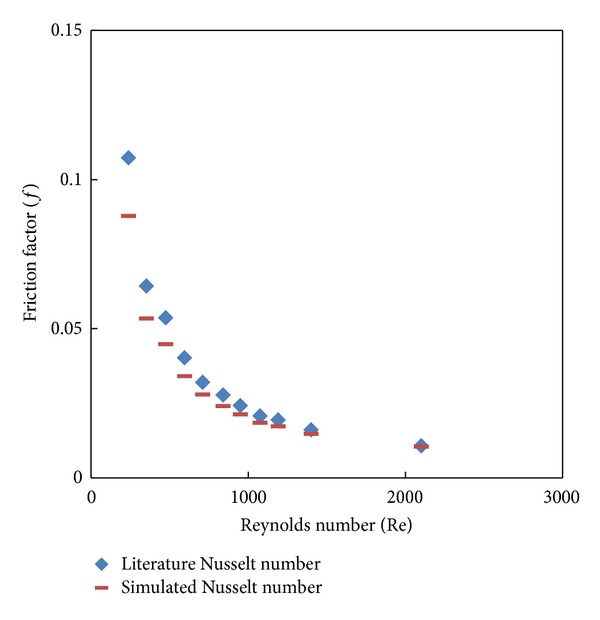
Plain tube simulated friction factor versus the literature data.

**Figure 6:**
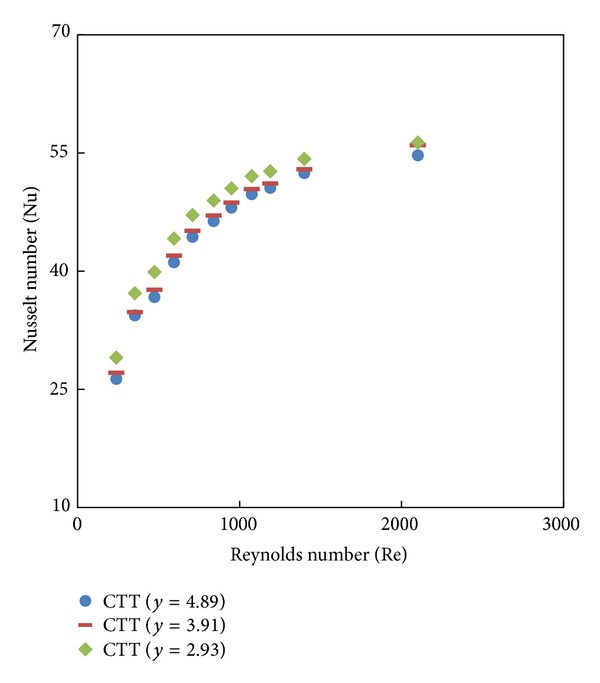
Simulated Nusselt friction factor of twisted tape with different twist ratios (*y*).

Testing of the grid independence was carried out to evaluate the effects of grid sizes on the accuracy of the simulated results. In this study, five mesh volumes were considered, 200132, 233228, 292655, 373825, and 390191 at laminar flow Re = 2000. It is observed that the accuracy of the result increases with increase in mesh number (nodes) and the accuracy became constant around 292655 cells for plain tube and tube fitted with classical twisted tape inserts, and hence final CFD simulations were done at this cell. The results of the Nusselt number and friction factor for plain tube simulation are validated with Sieder and Tate [25] correlations as shown in Figures 5 and 6. Apparently, present results reasonably agree well with these correlations with maximum discrepancy of ±8% in the Nusselt number and ±10% for friction factor.

### 7.2. Effect of Twist Ratio on Heat Transfer and Friction Factor

**Figure 7:**
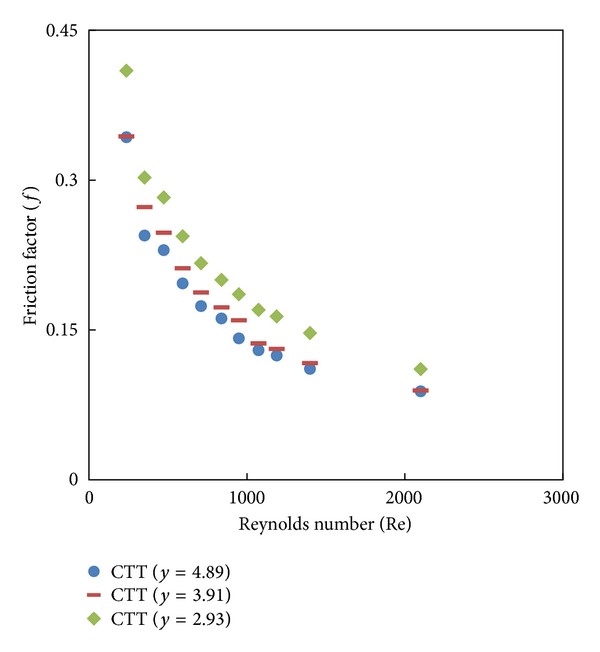
Simulated friction factor of twisted tape with different twist ratios (*y*).

The simulated data of the Nusselt number and friction factor and their variation with a Reynolds number of twisted tape inserts with twist ratios *y* = 2.93, 3.91, and 4.89 are shown in Figures 6 and 7.  Figure 6 indicates that the Nusselt number increases with increasing Reynolds number and the heat transfer rate is higher for the twist tape set than the plain tube, because of strong swirl flow in the presence of the twist tape. It is found that the heat transfer rate with the twist ratio *y* = 2.93 is higher than those from other ratios (*y* = 3.91 and 4.89), and this means that the turbulent intensity obtained from the lower twist ratio is higher than those of higher ratios (*y*).  Figure 7 shows the variation of friction factor with a Reynolds number for different twist ratios (*y* = 2.93, 3.91, and 4.89). The friction factor obtained from the tube with twisted tape insert is significantly higher than that from the plain tube. Moreover, the use of smaller twist ratio leads to higher tangential contact between the swirling flow and the tube surface. Therefore, the twisted tape with twist ratio (*y* = 2.93) has a maximum friction factor. 

### 7.3. Effect of Cut Depth on Heat Transfer and Friction Factor

**Figure 8:**
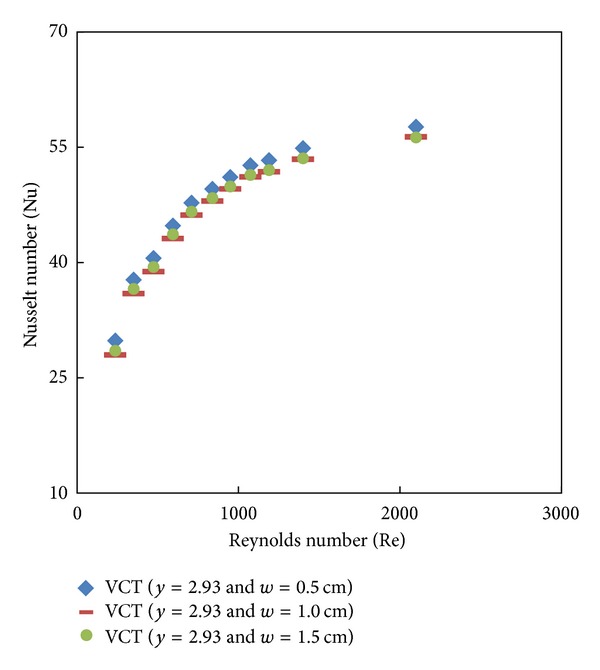
Simulated Nusselt number of V-cut twisted tape with different twist ratios (*y*) and different cut depths.

**Figure 9:**
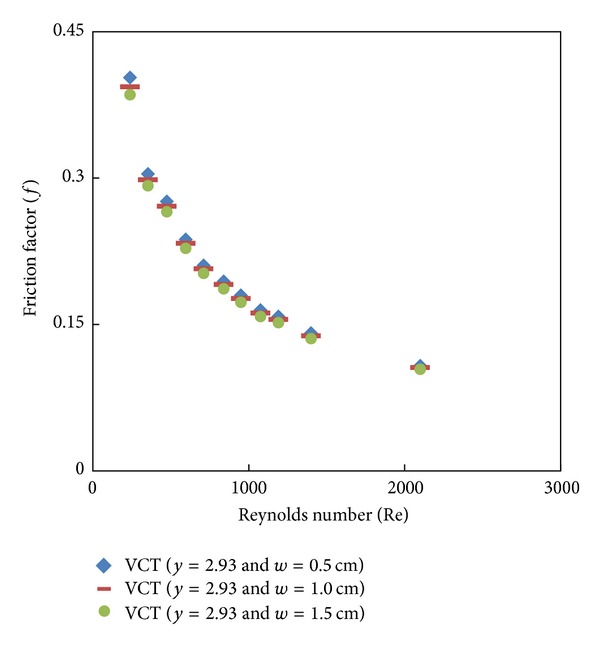
Simulated friction factor of V-cut twisted tape with different twist ratios (*y*) and different cut depths.

**Figure 10:**
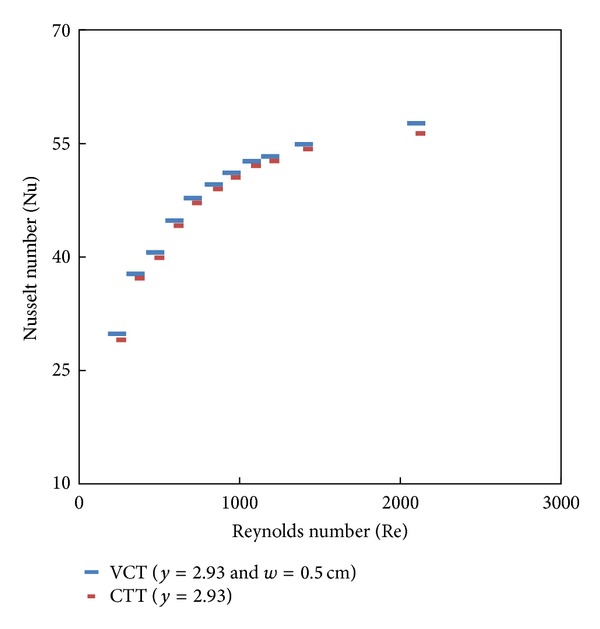
Simulated Nusselt number of classical and V-cut twisted tape inserts (*y* = 2.93) with cut depth *w* = 0.5 cm.

**Figure 11:**
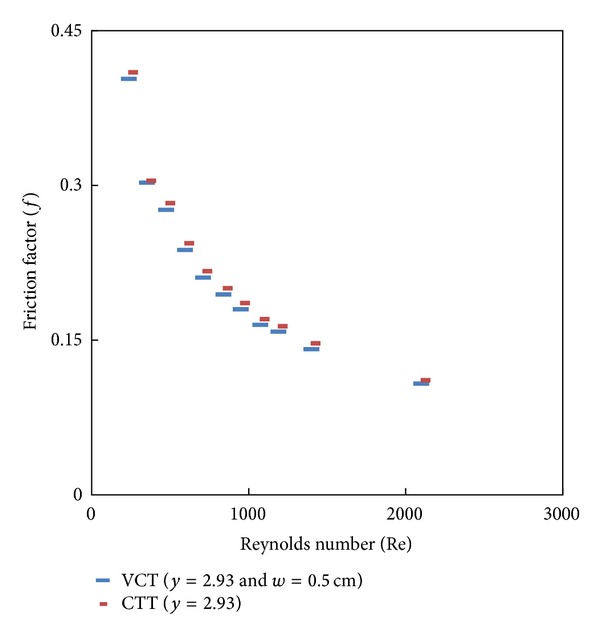
Simulated friction factor of classical and V-cut twisted tape inserts (*y* = 2.93) with cut depth *w* = 0.5 cm.

**Figure 12:**
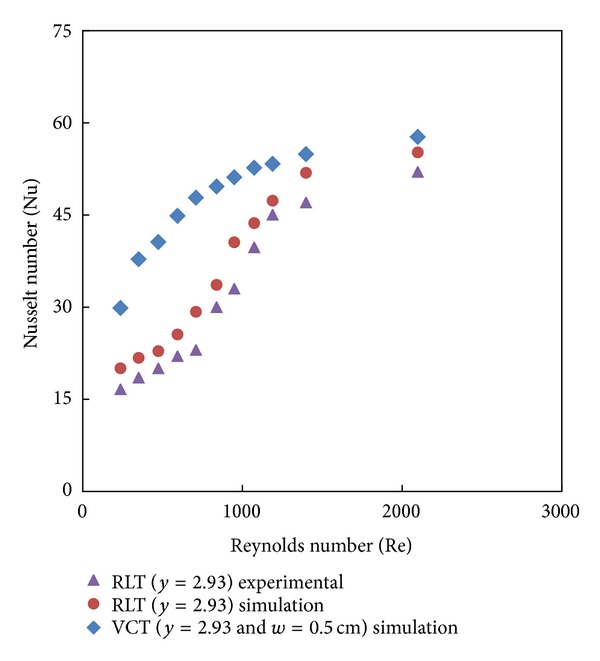
Simulated Nusselt number of V-cut twisted tape and RLT inserts.

**Figure 13:**
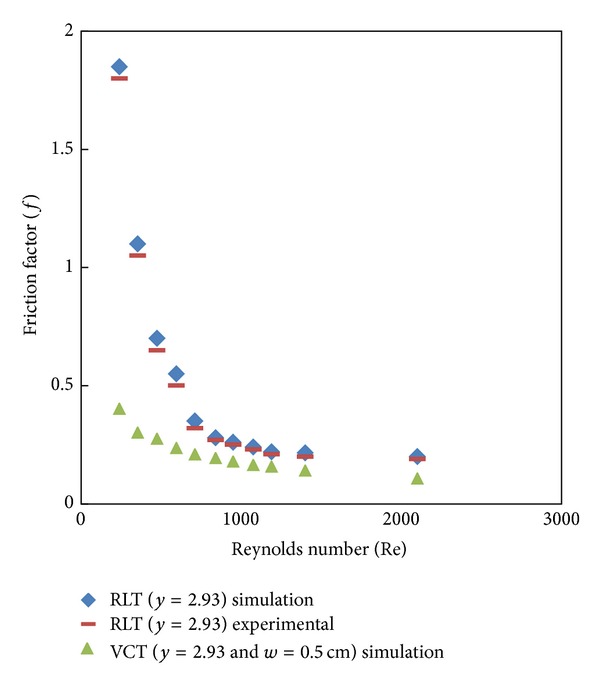
Simulated friction factor of V-cut twisted tape and RLT inserts.

Simulated data for the Nusselt number and friction factor and their variation with Reynolds number for V-cut twisted tape inserts with twist ratio *y* = 2.93 and cut depths (*w* = 0.5, 1, and 1.5 cm) is shown in Figures 8, 9, 10, and 11. For a given Reynolds number, Nusselt number and friction factor are increased with decreasing cut depth, and this is mainly due to the combined effects of common swirling flow by the twisted tape and turbulence generated by the alternative cuts along the edge of the twisted tape which leads to the destruction of the thermal boundary layer and creating better flow mixing between the fluids at the core and heating wall surface. Furthermore the simulated results of the Nusselt number and friction factor of V-cut twisted tapes with twist ratio *y* = 2.93 and cut depth *w* = 0.5 cm are compared with experimental simulated data on helical twist tape [21] as shown in Figures 12 and 13. It is found that the VCT offered an additional heat transfer enhancement with less friction factor.

## 8. Conclusion

CFD simulation for the heat transfer augmentation transfer and friction factor in a circular tube fitted with classical and V-cut twisted tape inserts with *y* = 2.93, 3.91, and 4.89 and cut depth *w* = 0.5, 1, and 1.5 cm in laminar flow conditions has been reported using FLUENT version 6.3.26. The results are concluded as follows.

- The values of the Nusselt number and friction factor for the tube with V-cut twisted tape are noticeably higher than the values for the plain tube and tube equipped with classical twisted tapes as well.
- Over the range of the Reynolds numbers considered, the tube equipped with the V-cut twisted tape *y* = 2.93 and *w* = 0.5 cm offered supreme heat transfer enhancement than the classical twisted tapes and other V-cut twisted tapes used.
- The simulated data for V-cut twisted tape *y* = 2.93 and *w* = 0.5 cm is compared with theoretical and experimental work of Right-left helical tape inserts (RLT) with twist ratio *y* = 2.93 at the same conditions. The results show that the proposed model of V-cut twisted tape offered 107% heat transfer enhancement with less friction factor. This configuration can be used for heat transfer augmentation.

## Figures and Tables

**Table 1:** Thermo-physical properties of materials.

Materials	Density (Kg/m^3^)	Specific heat (J/kg·K)	Thermal conductivity (W/m·K)	Viscosity (Pa·s)
Water	998.2	4182	0.6	0.001003
Steel	8030	502.48	16.27	—
Aluminium	2719	871	202.4	—

**Table 2:** Numerical values of the parameters used for simulations [12].

Mass flow rate (Kg/s)	Heat flux (W/m^2^)
0.003	240.03
0.005	340.77
0.006	459.15
0.008	563.38
0.01	479.85
0.0116	1001.57
0.0133	1363.26
0.015	1512.68
0.0166	1893.56
0.02	2130.05
0.03	2445.25

## Nomenclature

*E*: Energy component in energy equation
*F*: Force component in momentum equation, N
*f*: Fanning friction factor
*g*: Acceleration due to gravity, m/s^2^
*k*_eff_: Thermal conductivity in energy equation, W/m K
*m*: mass flow rate of fluid, kg/s
Re: Reynolds number based on internal diameter of the tube, dimensionless
Nu: Nusselt number, dimensionless
*p*: Pressure component in momentum equation, N/m^2^
*S*_*m*_: Accumulation of mass, Kg
*S*_*h*_: Accumulation of energy, J
*T*: Temperature, °C.
*v*: Velocity component in momentum equation, m/s
*y*: Twist ratio (length of one twist (360°)/diameter of the twist), dimensionless.

### Greek Symbols

*ρ*: Density component in governing equations
${\vec{\tau }}_{\text{eff}}$: Stress component in momentum equation, N/m^2^.
